# Surgical Tolerability and Frailty in Elderly Patients Undergoing Robot-Assisted Radical Prostatectomy: A Narrative Review

**DOI:** 10.3390/cancers14205061

**Published:** 2022-10-16

**Authors:** Yuta Yamada, Satoru Taguchi, Haruki Kume

**Affiliations:** Department of Urology, Graduate School of Medicine, The University of Tokyo, Bunkyo-Ku, Tokyo 113-8655, Japan

**Keywords:** robot-assisted radical prostatectomy (RARP), prostate cancer, elderly, frail, frailty, surgical tolerability

## Abstract

**Simple Summary:**

Life expectancy in Western countries and East Asian countries has incremented over the past decades, resulting in a rapidly aging world, while in general, radical prostatectomy (RP) is not recommended in elderly men aged ≥75 years. Together with the evolving technique of robotic surgeries, surgical indications for RP should be reconsidered in ‘elderly’ and ‘frail’ men, since this procedure has now become one of the safest and most effective cancer treatments for prostate cancer. One important element to determine surgical indications is surgical tolerability. However, evidence is scarce regarding the surgical tolerability in elderly men undergoing robot-assisted radical prostatectomy (RARP). In this review, we focused on the surgical tolerability in ‘elderly’ and/or ‘frail’ men undergoing RARP, with the intent to provide up-to-date information on this matter and to support the decision making of therapeutic options in this spectrum of patients.

**Abstract:**

Robot-assisted radical prostatectomy (RARP) has now become the gold standard treatment for localized prostate cancer. There are multiple elements in decision making for the treatment of prostate cancer. One of the important elements is life expectancy, which the current guidelines recommend as an indicator for choosing treatment options. However, determination of life expectancy can be complicated and difficult in some cases. In addition, surgical tolerability is also an important issue. Since frailty may be a major concern, it may be logical to use geriatric assessment tools to discriminate ‘surgically fit’ patients from unfit patients. Landmark studies show two valid models such as the phenotype model and the cumulative deficit model that allow for the diagnosis of frailty. Many studies have also developed geriatric screening tools such as VES-13 and G8. These tools may have the potential to directly sort out unfit patients for surgery preoperatively.

## 1. Introduction

In elderly patients, several clinical elements construct the background of decision-making for the treatment of prostate cancer (PCa). One of the important elements is the rapid acceleration of global aging. Life expectancy is prolonged in the United States, as well as in European and East Asian countries. For example, the life expectancy at birth in the United States was 74.12 years in the year 2000 but was elevated to 76.28 years in 2019 [1]. Similar trends were observed in most of the European and East Asian countries [1]. Notably, in some countries such as Portugal, Spain, and Ireland, the life expectancy at birth has been prolonged by more than 5 years during the last decade [1]. 

We also know that older men with PCa have a higher chance of harboring higher-grade tumors, advanced stage, and worse prognosis [2]. Recent statistics show that patients aged ≥60 years are burdened by a higher probability of developing invasive Pca [3]. Additionally, Pca is the third and second leading cause of death in patients aged 60–79 and ≥80 years, respectively [3]. 

Another concern is the time-consuming and complex flow chart of the current guidelines [4,5,6]. For instance, in the NCCN guideline, surgeons would have to check the recommended life expectancy for the treatment depending on the risk group of Pca. Then, a rough estimate of life expectancy, in general, is investigated by searching the data of the WHO [7] or referencing the appropriate statistics [8]. Finally, the life expectancy is adjusted by combining the information of health status from three categories [4]. However, the method on how to evaluate the health status is up to the surgeons. In addition to this, the biggest concern is the insufficient amount of evidence to strongly provide a recommendation for treatment in elderly men. This is partly because elderly men are not usually eligible for randomized controlled studies or clinical trials [9]. In this context, urologists may have to make their own clinical decision in individual cases and carry out their practice by subjectively combining the health status to of surgical indication or even make decisions according to the evidence based on men of younger age. 

One idea to distinguish ‘fit’ from ‘unfit’ patients regarding surgical indication is to introduce the idea of ‘frailty’. Although there are some geriatric assessment tools to evaluate patients with ‘frailty’, one is not superior to another. Therefore, to this day, the most effective tool to discriminate between ‘fit’ and ‘unfit’ patients for surgery is not universal. Additionally, there are also tools to predict life expectancy, but those do not fully meet the surgeon’s trust, since the calculation is rather a rough estimate. Consequently, there are still many urologists that depend more on chronological age or the surgeon’s experience for the decision-making of cancer treatment.

We must also bear in mind the paradigm shift of the prevalence of robotic surgery. The surgical technique has evolved for the surgical treatment of PCa over the couple of decades with the introduction of RARP. Surgical robots are multi-armed with joints that provide smooth motion and are capable of reducing the tremor of surgeons. It also has a three-dimensional magnified view that provides an excellent view of the surgical field. Given the advantage that RARP embraces, the surgical indication may be extended to patients that would have been treated otherwise before the RARP era.

Taken together, these elements lead to complex and controversial decision making in the management of PCa. In this review, we evaluate the previous publications on elderly and frail patients undergoing RARP, with a particular focus on surgical tolerability, and address future perspectives in this field.

## 2. Surgical Tolerability in ‘Elderly’ Patients

### 2.1. The Definition of ‘Elderly’

Conventionally, the WHO uses ‘65 years of age and over’ as the definition of ‘elderly’ [10], and in fact, most pension schemes worldwide use a cutoff value of around 65 years for eligibility [11]. The Organisation for Economic Co-operation and Development (OECD) also defines ‘elderly’ as 65 years and over [12]. However, the definition of the term ‘elderly’ itself is not universally applicable and also changing, potentially differing across countries. For instance, in Japan, ‘elderly’ was defined as having a chronological age of 65 years or older until 2017, when the Joint Committee of Japan Gerontological Society and the Japan Geriatrics Society announced that ‘elderly’ should be redefined as ages 75 years and older [13]. Their proposition was based on the ‘rejuvenation’ phenomenon in the elderly population in recent years [13]. Specifically, physical functions including gait speed and grip strength were significantly greater in the fiscal year 2002 than those in 1992 [13]. In addition, the International Society of Geriatric Oncology (SIOG) updated its recommendations on prostate cancer management in older patients in 2019 [14]. According to this expert consensus, it was stated that men aged 75 years and older with prostate cancer should be managed according to their individual health status by using geriatric assessment tools instead of making decisions by chronological age.

### 2.2. Evidence on the Feasibility of RARP in Elderly Men Aged ≥75 Years

Alongside the changes in the concept of the term ‘elderly’, increasingly more published data are presented for men aged ≥75 years undergoing RP (Table 1). To note, no randomized controlled trial (RCT) or prospective study has compared the complication rates between the elderly and younger population. However, six retrospective studies report perioperative outcomes in patients undergoing RP, of which, three showed a direct comparison of functional outcomes and complication rates between elderly and younger men (Table 1).

One of the first studies to evaluate the surgical, oncologic, and functional outcomes in men aged ≥75 years was reported by Labanaris et al. [15]. This retrospective study reported higher rates of minor complications in the elderly group (15.5% vs. 11.4%), although it was compared to that of the entire cohort. We previously investigated the perioperative, oncologic, and functional outcomes of RARP in men aged ≥75 years in comparison with younger men [16]. Although there were no significant differences in terms of complication rates, the duration of hospitalization was longer in the elderly group. Specifically, Clavien-Dindo grade ≥3 complication rates were 2.6% and 4.3% with no statistical difference between men aged ≥75 years and younger, respectively [16]. Togashi et al. reviewed 752 patients who underwent RARP and compared the results of self-reported questionnaires among three groups on the basis of age (age <70, 70–74, and ≥75 years) [17]. The oncologic outcomes and pad-free rates were similar among the groups. Regarding complication rates, Clavien-Dindo grade 3 complication rates were 0.6%, 0%, and 0% in men aged <70 years, 70–74 years, and ≥75 years, respectively [17].

The most recent studies included a large population of elderly patients. Ko et al. reviewed the clinical records of 1110 subjects aged ≥75 years, of which 883 and 227 underwent RARP and RT, respectively [18]. This study showed that overall mortality (OM) was associated with diabetes and cancer-specific mortality and was inversely associated with low-risk PCa, but not the type of treatment (RP or RT) after using the inverse probability of treatment-weighting (IPTW) modeling [18]. Leyh-Bannurah et al. investigated clinical outcomes in 669 patients aged ≥75 years and 8268 patients aged <70 years [19]. Thirty-day complication rates were similar between men aged ≥75 years and <70 years. Erectile function and biochemical recurrence (BCR) rates were lower in men aged ≥75 years after propensity score matching (27% vs. 56%, 77% vs. 85%, respectively) [19].

There is only one retrospective study showing data from men aged ≥80 years, although this study was not conducted in a comparative design [20]. This study showed the perioperative and postoperative complications after RARP in 46 patients aged ≥80 years [20]. Nine patients had postoperative complications, of which, two were Clavien-Dindo grade ≥3. The authors noted that RARP represents a feasible treatment option in well-selected octogenarian men.

Most of these studies showed no significant increase in complication rates in the elderly population, although this spectrum of men may have been well selected. These studies suggest that RARP may be feasible in elderly men when the target population is well-selected, and it may also suggest the need for indications to select patients for RARP. Evidence is scarce regarding this issue with no RCT studies comparing the surgical outcomes between the elderly and younger men.

### 2.3. Comparison of Treatment Options in Elderly Men

#### 2.3.1. Cancer Control

Several landmark studies compared the oncologic outcomes among RP, RT, and observational treatment options [21,22,23]. The ProtecT trial compared active monitoring, RP, and external-beam RT (EBRT) for the treatment of clinically localized PCa and found no statistical significance in cancer-specific mortality among treatment options [21,22]. Although there was a sub-analysis assessing the impact on men over 65 years old, patients included in the study were all under 70 years old and therefore may not have shown the true nature of characteristics in the elderly population. Another RCT assigned 695 men to either RP or watchful waiting and compared mortality with a median follow-up of 23.6 years [23]. The cumulative incidences of death from PCa at 23 years were 19.6% and 31.3% for RP and watchful waiting, respectively. This study also performed a sub-analysis in men aged <65 vs. ≥65 years and found that more benefits regarding mortality and metastasis were likely to be provided by RP in the younger group. A large prospective study by Nepple et al. compared PCa-specific mortality among 10,361 patients undergoing either RP, EBRT, or brachytherapy (BT) with a median follow-up of 7.2 years [24]. Age was a significant factor in the univariate analysis but did not remain statistically significant in the multivariate analysis predicting PCa-specific mortality.

Unfortunately, there are no RCTs evaluating the comparison of treatments in an elderly population. Notably, one large retrospective study including 10,563 men aged ≥75 years with cT2 localized PCa showed that RP was superior to RT in overall survival and cancer-specific survival after propensity score matching (hazard ratio (HR) = 0.54, 95% confidence interval (CI) = 0.47–0.62, and HR = 0.30, 95% CI = 0.20–0.45) [25]. Interestingly, a sub-analysis in the study showed that for patients with Gleason score (GS) = 7, RP provided a higher risk decline of overall death when compared with RT treatment [25].

#### 2.3.2. Complications after Treatment in Elderly Men with PCa

Comparing the risk of complications as well as cancer control is essential in treatment optimization, especially for elderly men, since tolerability may have deteriorated in this segment of the population and intervention may accelerate the vulnerability. For instance, androgen deprivation therapy (ADT) is known to increase the risk of bone fracture [26], diabetes [27,28], and cardiovascular disease [29,30], although there are some ways to mitigate these concerns [31]. Since testosterone has a neuro-protective effect including the improvement of energy metabolism and reduction of oxidative stress in neurons [32], ADT may deteriorate cognitive function [33] and may increase the risk of dementia as well [34]. Low serum testosterone level also correlates positively with lean body mass, which can be calculated by subtracting the weight of all the fat from the total weight of the body [35]. Interestingly, androgen deprivation therapy may selectively decrease lower-limb muscle function mediated by reduction of the iliopsoas and quadriceps force by 14% and 11%, respectively [36]. Another study investigating sarcopenia during ADT for PCa revealed that men of age ≥70 years had a significantly greater reduction of lean body mass than that of younger men [35]. Taken together, ADT may accelerate the deterioration of mobility in elderly men undergoing ADT for PCa.

Radiation therapy used in the treatment of PCa varies from brachytherapy (BT) to external beam radiation therapy (EBRT). In general, urinary obstruction and urethral stricture are the most common severe urinary toxicities [37]. The incidence rate of urethral stricture after EBRT is <7% with <5 years of follow-up and increases to 10–18% with 5–10 years of follow-up [38]. This means that the longer the life expectancy at the time of EBRT therapy, the higher the risk of having this complication over a lifetime. Another concern in patients undergoing RT is hematuria. The 5- and 10-year incidences of macrohematuria are 5% and 8%, respectively, in patients undergoing RT [39]. As the use of anticoagulants may increase in elderly men due to an increasing incidence of atrial fibrillation and venous thromboembolism [40] in the elderly population, they might be at more risk of suffering macrohematuria after RT. 

In general, patients who undergo RP are more likely to suffer complications involving urinary incontinence (UI) and erectile dysfunction (ED) when compared with other treatment modalities such as RT with or without ADT [41,42]. However, a recent propensity-score-matched study comparing complication status between RP and high-dose intensity-modulated radiotherapy (IMRT) combined with long-term hormone therapy (HT) showed otherwise with respect to erectile dysfunction in patients with age ≥80 years [43]. This retrospective cohort included 659 patients with high-risk PCa and was conducted by propensity score matching of a 1:2 ratio based on positive surgical margin status (277 patients for RP and 382 patients for IMRT + HT) and compared acute and chronic complications after treatments [43]. The approach of RP was not shown, and therefore the number of cases performed in robotic procedures is unknown. In addition, patients in the IMRT + HT group received 1.5–3 years of long-term HT in combination with IMRT. The rates of erectile dysfunction were significantly higher in the IMRT + HT group at 3 months and 1 year after treatment [43]. Benign prostatic hyperplasia (BPH) symptoms were more frequently observed in the IMRT+HT group at any time points throughout the study (days 1–90, days 91–365, 2 years, 3 years, 4 years, and 5 years after treatment) [43]. On the contrary, UI was more frequently observed in the RP group throughout all time points. After 5 years, common chronic complications were BPH symptoms and UI (RP vs. IMRT+HT: 17.7% vs. 29.6% and 10.5% vs. 5.5%, respectively) [43]. Notably, the occurrence rate of hernia was also higher in the RP group, although the type of hernia was not mentioned. Interestingly, the rate of impotence was comparable between the two groups after 2–5 years but even worse in the IMRT + HT group in the acute and subchronic phases after treatment [43]. 

A large population-based cohort study including a total of 32,465 patients undergoing either RP or RT reported the incidence of complications other than UI or erectile dysfunction [44]. The five outcomes implemented in the study were (1) necessity of hospital admission to manage a treatment-related problem; (2) minimally invasive urological procedure (e.g., cystoscopy); (3) rectal or anal procedure (e.g., endoscopy); (4) open procedure related to the urinary tract, rectum, and anus; and (5) development of secondary malignancy. Interestingly, RT showed higher risks in all outcomes except for minimally invasive urological procedures (HR 0.66 (95% CI: 0.63–0.69), *p* < 0.0001). As for hospital admission, HR in the RT group compared with the RP group incremented every year starting from the first year, which was 0.86 and to 10.8 at 5 years. Similarly, HR in the RT group for the open surgical procedure was also incremented every year, starting from 1.15 in the first year to 3.68 in 5 years. Regarding rectal or anal procedure and secondary malignancy, HRs were 2.72 (95% CI: 2.40–3.08; *p* < 0.0001) and 2.08 (95% CI: 1.48–2.91), respectively, in favor of RP. Notably, the study did not measure repeat complications or management and time to the first complication was used for analyzing the data. Assumably, multiple procedures such as coagulation of bleeding from the bladder or the prostate, as is often observed in cases treated with RT, were not taken into account. Since the median ages of patients undergoing RP and RT were 62 and 70 years, respectively, the population of the cohort was characterized as rather younger aged. Therefore, future studies are expected to show the results of comparison among treatments in the older population.

## 3. Frailty and Surgical Tolerability

### 3.1. Age-Related Changes

Skeletal muscles are known to form the largest tissue constructing the body and also provide three major functions such as maintaining posture/locomotion, providing protein and amino acids, and producing body heat [45]. However, skeletal muscles significantly decrease with age, commonly beyond the age of 60 years, resulting in a reduced maximum voluntary force of contraction in the proximal and distal muscles [45,46,47]. This reduction of muscle mass and strength is known as sarcopenia, which correlates with an increased risk of frailty and falling [48]. Aging also decreases the level of testosterone (T) production, and this accelerates sarcopenia [48] and osteoporosis [49]. Low levels of T are also associated with atherosclerotic plaques and endothelial dysfunction that affect the cardiac muscles and vascular smooth muscles resulting in reduced cardiac power output or incrementing risks of coronary or cerebral disease [45]. Especially in elderly patients, arterial stiffening, or arteriosclerosis, is associated with a reduction of cognition and age-related pathology, including Alzheimer’s disease and dementia [50]. 

### 3.2. The Definition and Models of ‘Frailty’

The definition of ‘frailty’ is a state of increased vulnerability to poor resolution of homeostasis following stress, which increases the risk of adverse outcomes including falls, delirium, and disability [51,52]. Although the criteria of ‘frailty’ vary, the two principal models of ‘frailty’ are the phenotype model suggested by Fried et al. known as the ‘Fried frailty criteria’ and the cumulative deficit model represented by the Canadian Study of Health and Aging (CSHA) Frailty Index [52,53,54]. The former phenotype model was initially developed by analyzing the data from the Cardiovascular Health Study (CHS) that contained 5210 men and women aged 65 years and older [53]. The phenotype model was based on unintentional weight loss, self-reported exhaustion, low energy expenditure, slow gait speed, and weak grip strength. Patients with three of the five factors were defined as ‘frail’, with one to two factors as ‘pre-frail’, and no factors as robust [53]. To note, this model does not include content regarding cognition and mental health. 

The CSHA Frailty Index (FI) represents the latter cumulative deficit model that was developed from the CSHA, which was a 5-year prospective cohort study initially including 10,263 people aged 65 years and older [54]. The CSHA-FI was based on a count of 70 clinical deficits from the CSHA clinical assessment [54]. Later, subsequent studies suggested models that were based on a reduced number of clinical deficits; modified FI-11 (mFI-11) and modified FI-5 (mFI-5) [55,56]. The mFI-11 was calculated using 11 factors such as functional status, history of diabetes, respiratory problems, congestive heart failure, myocardial infarction, cardiac problems, arterial hypertension, delirium, history related to cognitive impairment or loss, cerebrovascular problems, and history of stroke/decreased peripheral pulses [55,56]. The mFI-5 consists of functional status, diabetes, history of chronic occlusive pulmonary disease, history of congestive heart failure, and hypertension requiring medication [56]. The mFI-5 showed strong correlations with mFI-11 in any type of surgery, and it also showed acceptable predictive value for the postoperative complication, unplanned 30-day readmission, and mortality regarding general surgery [56].

Although the Fried phenotype model and cumulative deficit model overlap in the area of physical functional status, the latter model showed greater discrimination for patients with moderate and severe frailty [52,57]. In addition, it is more likely to reflect the idea of Comprehensive Geriatric Assessment (CGA).

Frailty also has close links with sarcopenia [48,58]. Although the concepts of frailty and sarcopenia are evolving, frailty is focused on a framework to detect people with a high risk of disability. On the other hand, sarcopenia is considered a muscle failure or muscle insufficiency that may lead to physical frailty [58]. In this context, both concepts overlap in the physical element [58].

### 3.3. Evaluation of Frailty: Comprehensive Geriatric Assessment and Screening Tools

The gold standard for assessing health status is the CGA [59]. The CGA is a multifaceted evaluation method that can detect physical, functional, and mental deficits of the patient [60]. According to the latest recommendation of the International Society of Geriatric Oncology (SIOG) Taskforce, the geriatric domains that should be assessed in CGA are functional status, comorbidity, cognition, mental health status, fatigue, social status and support, nutrition, and geriatric syndromes (e.g., dementia, delirium, falls, incontinence, osteoporosis, polypharmacy, and sarcopenia) [61]. It is known that the use of CGA not only improves cognitive function and reduces disability progression, as well as reducing the risk of falls, unplanned hospitalization, and nursing home admission, but that it may also improve mortality, although this may be limited to the younger-age subjects [52,62,63]. On the contrary, the greatest disadvantage of CGA is the time-consuming assessment and necessity of multiple types of expertise, including geriatrists, urologists, and potentially even physical therapists or physicians. On this ground, screening tools before implementing CGA were developed. 

The Vulnerable Elders Survey-13 (VES-13) and Geriatric 8 (G8) are some of the screening tools other than Fried frailty criteria [64,65,66]. Both tools can be self-reported or administered by a nurse with no expertise in geriatrics and can be completed in about 5 min [67,68]. In elderly patients with cancer, the sensitivity and specificity of predicting frailty on CGA were as follows: 68% and 78% for VES-13, 87% and 61% for G8, respectively [66].

VES-13 was developed to identify community-dwelling vulnerable older people at the risk of death or functional decline [64]. It contains 13 items that add up to a maximum of 10 points. Subjects with scores of ≥3 points had 4.2 times the risk of death or functional decline over a 2-year period [64]. This tool predicts mortality in several cancer types including gastrointestinal cancer [69], colorectal cancer [70], and prostate cancer [71]. Notably, in patients with prostate cancer who receive ADT, the sensitivity and specificity of VES-13 predicting impairment were 72.7% and 85.7%, respectively, when compared with CGA [67]. 

G8 consists of eight items that cover food intake, weight loss, mobility, neuropsychological problems, body mass index, polypharmacy, self-perceived health status, and age [65]. G8 scores range from 0 to 17, and most studies use 14 or 11 points as the cutoff line [72,73,74,75]. With the cutoffs of ≤14 as abnormal G8 scores, the G8 scores had a 65.2% sensitivity and 95.7% specificity for detecting vulnerability in PCa patients [76].

### 3.4. Association between ‘Frailty’ and Complication Rates in RARP Patients

Previous retrospective studies have shown comparable outcomes in men aged ≥75 years (Table 1). However, these studies have inevitable bias generated from the retrospective design. Specifically, the cohort with elderly men undergoing RARP may be well selected in terms of surgical tolerability. Under the circumstances where there are no RCTs to confirm the safety of recommending RARP in this set of patients, it may be important to find a way to discriminate against patients associated with ‘surgical intolerability’. Frailty has grown attention from surgeons since this spectrum of patients may be the center standpoint of patients with ‘surgical intolerability’. 

Evidence on perioperative outcomes of RARP in association with frailty is shown in Table 2. Six retrospective studies were identified, of which, four studies consisted of a pure population of RARP patients [77,78,79,80,81,82]. Notably, three studies used G8 to detect frailty. 

Rosiello et al. explored postoperative outcomes in 91,618 patients treated after RP [78]. Overall, 12,185 (13.3%) patients were identified as being frail. Frail patients had higher rates of overall and major complications (16.6% vs. 8.6% and 4.9% vs. 2.6%, respectively). Interestingly, of these frail patients, approximately 86% neither exhibited a body mass index (BMI) ≥ 30 nor Charlson Comorbidity Index ≥ 2, suggesting that frail patients are at risk of postoperative outcomes that cannot be predicted by other factors, such as BMI or comorbidities. 

Another retrospective study including 23,104 patients undergoing RARP investigated the association between frailty and Clavien–Dindo grade 4 (CDIV) complications in RARP patients [77]. A modified frailty index score using 15 variables was used in the study. Men with modified frailty index (mFI-15) score of ≥3 had a high odds ratio of 12.1 (CI: 2.80–52.3) in comparison with non-frail patients [77]. Patients with higher mFI-15 scores were also likely to have higher rates of wound disruptions, bleeding transfusions, and 30-day mortality, as well [77]. Interestingly, they suggested a combined variable of mFI-15 and the American Society of Anesthesiology classification to predict 30-day mortality for RARP patients (C-index 0.709) [77]. 

Momota et al. investigated the G8 scores in patients with PCa and found that G8 scores were higher in RARP than in RT or ADT-alone treatment groups, indicating surgeons’ potential selection bias of patients undergoing certain types of treatment [79]. They also evaluated the Fried phenotype criteria and found that the compatibility of frailty between G8 ≤ 13 and Fried phenotype criteria (≥3) was acceptable (Cohen’s kappa = 0.268, *p* = 0.007). 

A large retrospective cohort study assessed the association between simple 5-item frailty index (5-iFI) scores and surgical outcomes of RP [80]. The 5-iFI was based on the following five indicators: chronic obstructive pulmonary disease or pneumonia, congestive heart failure, dependent functional status, hypertension, and diabetes. The study included 15,546, 14,541, and 3,556 patients with simple 5-item frailty index (5-iFI) scores of 0, 1, and ≥2, respectively [80]. Unsurprisingly, patients with ages >65 years were more likely to have higher 5-iFI scores. In addition, 5-iFI score ≥ 2 showed increased odds ratios of 1.66 (CI: 1.31–2.11) and 1.85 (CI: 1.39–2.46) in patients with Clavien–Dindo grades ≥3 and ≥4, respectively [80]. Unfortunately, the type of approach regarding RP was not mentioned in this study. 

The FRART-PC Study developed a nomogram that included G8 scores to predict ‘surgical indication’ for PCa on the basis of the data of 479 patients with localized PC who were treated with RARP or RT [82]. In the study, surgical indication was analyzed with respect to association with G8 scores and other factors after it was determined by the surgeon according to the presence of comorbidities and/or patient preference. Therefore, the nomogram in this study may only predict the ‘surgical indication’ determined by the surgeon and patient preference and thus does not predict the true surgical indication that should be determined from the perspective of complication rates or morbidity. Moreover, the variables regarding comorbidities were used in both the nomograms and the predicted binary outcome of ‘surgical indication’, which may generate some limitations regarding the study design. 

The impact of the G8 tool on quality of life and lower urinary symptoms seems to be insignificant. A longitudinal prospective study was conducted on 118 patients undergoing RARP for 12 months and investigated the G8 scores to classify patients into either frail (G8 ≤ 14) or non-frail (G8 > 14) groups [81]. Health-related quality of life (HRQOL) and lower urinary tract symptoms (LUTS) were also assessed but unfortunately had no significant association with frail [81].

## 4. Patient Preference

Previous literature showed that older patients associated with cancer have been both under-investigated and under-treated [83]. A systematic review revealed that factors for declining cancer treatment included concerns regarding discomfort of the treatments, fear of side effects, and transportation difficulties, while factors for accepting cancer treatment were convenience and success of treatment, the necessity of treatment, trust in the physician, and following the physician’s recommendation [83]. Additionally, the expectation of complete tumor removal is a strong factor that affects decision making on selecting RP [84,85].

Togashi et al. reviewed 752 patients who underwent RARP and compared the results of self-reported questionnaires among three groups on the basis of age (age < 70, 70–74, and ≥75 years) [17]. The oncologic outcomes and pad-free rates were similar among the groups. Nevertheless, the ratio of patients attaining full satisfaction (score = 100) quantified by the Expanded Prostate Cancer Index Composite (EPIC) questionnaire at 12 months was significantly higher in the ‘ages ≥ 75 years’ group than in the ‘ages < 70 years’ group (27% vs. 15%, respectively) [17]. The authors commented that younger patients experienced a certain degree of deterioration of health-related quality of life after RARP compared with the baseline, while this deterioration was not observed for older patients [17].

According to a study assessing the impact of preoperative sexual function level and patient preference on receipt of active surveillance (AS) in patients with low-risk cancer, 52.6% of the men showed a strong preference for preserving sexual function, although older men were less likely to do so [86].

In a total of 509 men with PCa, Paudel et al. assessed the Personal Patient Profile-Prostate (P3P), a self-reported questionnaire that collects information on the bladder, bowel, or sexual problems and their influence on the decision making of treatment modalities [87]. The authors noted that men who suffered bladder, bowel, and sexual problems as having ‘a lot of influence’ on their treatment-decision chose AS, and further suggested that many men in the elderly population who have less concern over these problems may not choose AS [87]. 

Although the urologists’ recommendation or decision aid was the most important factor that influenced patients’ decision to choose certain treatment modalities such as active surveillance [88,89], recommendation of treatment may not always align with the patient’s preference. Especially in the elderly population, cognitive problems may occur involving acceptance of recommended treatment, and travel distance to the treatment facility may be an extra burden. Further, the partner may have a great influence on the patient’s preference [90,91]. In a study evaluating the self-reported questionnaires completed by the partners of patients with PCa, 88% of the partners reported active involvement throughout the decision-making process [90]. Unfortunately, this would not be the case for some patients in the elderly population without a partner. 

The accessibility to robotic surgery may also be involved in the decision making of undergoing RARP. An interesting study by Sugihara et al. showed a 13% reduction in RP caseload in non-robot hospitals, whereas a 101% gain in caseload was observed in hospitals with surgical robots [92]. Another study by Muralidhar et al. investigated the association between travel distance and choice of treatment [93]. Interestingly, 53.3% of the urban patients preferred RT, compared with 47%, 43.6%, and 33.8% of those living 5–10, 10–15, or >15 miles away from the treatment facility, respectively [93]. On the contrary, rural patients were less likely to choose RT the farther they lived from the treatment facility [93]. Distance to the treatment facility may be a significant burden to elderly patients and influence the decision making of treatment modalities. Presumably, RT may be an infeasible option for certain patients who have difficulty accepting daily treatment for over several weeks [93].

On this ground, urologists must bear in mind the total situation of the patient’s environment and not make a dogmatic decision on treatment options. In addition, urologists should provide adequate clinical information to patients, since patients may not have sufficient knowledge to determine treatment options that may lead to decision-regret post-operatively.

## 5. Present Recommendation of Treatment in Elderly Patients with PCa and Future Perspectives

**Figure 1:**
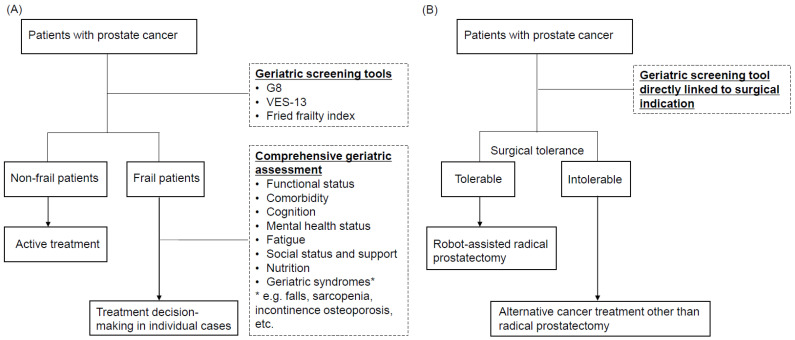
Flowcharts showing recommended use of geriatric tools in decision-making of treatment for prostate cancer. (**A**) Use of geriatric screening tools and comprehensive geriatric assessment in status quo. (**B**) Future perspective of direct use of geriatric screening tool to determine surgical tolerability.

Surgical tolerability should be determined by the biological age, specifically when the type of surgery is less invasive such as the RARP procedure. The word ‘life expectancy’ is a troublesome term. It is very difficult to predict one’s life expectancy at a certain point in time in individual cases. The NCCN guideline tries to resolve this by implementing additional adjustments on the basis of three categories (best quartile of health-add 50%, worst quartile of health-subtract 50%, and middle two quartiles of health-no adjustment) [4]. However, this assessment depends on the clinician’s evaluation, and the method of this classification is vague. This is because the evidence is scarce on what type of geriatric assessment tools or predictors of health status should be used for surgical indications for RARP. Although implementing both geriatric screening tools and CGA to screen out ‘unfit’ patients for surgery seems to be a recommended method in the status quo (Figure 1A), future studies are required to demonstrate the type of clinical tools that can directly predict the risks of RARP in elderly men (Figure 1B). In this way, urologists could proceed oncologic practice without consuming time for investigation on frailty. Additionally, urologists would have more confidence in their treatment recommendations and patients can easily choose a treatment option. Geriatric screening tools that can directly determine the surgical tolerability may alternatively replace the role of life expectancy in future clinical guidelines. 

## Figures and Tables

**Table 1:** Previous studies evaluating perioperative outcomes of radical prostatectomy in men aged ≥75 years.

First Author, Year	Study Design	Patient Selection	No. Pts.	f/u (mo)	Findings
Labanaris, 2012 [15]	R, C	cT2: 74%, cT3: 26% ≥75 years vs. entire cohort	N45 vs. N2000	17	Similar continence and potency rates at 12mo, lower BMI, GS, RM.
Yamada, 2020 [16]	R, C	cT1c 78%, cT2-3 22% ≥75 years vs. <75 years	N46 vs. N568	34	Worse trend in duration of hospitalization. Similar CSS, urinary continence, blood transfusion rates, and complication rates.
Togashi, 2021 [17]	R, C	≥75 years vs. 70–74 vs. <75 years	N469 vs. N216 vs. N74	47	No significant differences among groups regarding G8, sFI, pad-free rates, BCRFS, CSS, OS, and complication rates.
Ko, 2021 [18]	R, C, IPTW	All ≥75 years; cN0M0 RARP vs. RT	N883 vs. N227	74	Similar CSS and OS (83.4% of RT were with ADT). DM and low-risk PCa were associated with OS.
Shahait, 2021 [20]	R	All ≥80 years; cN0M0	N46	NA	Mean length of stay: 1.21 days, postoperative complication occurred in 9 (7 were Clavien 1–2, 2 were Clavien ≥3); continence rates at 3 and 12 months were 68.4% and 84.8%, respectively.
Leyh-Bannurah, 2022 [19]	R, C, PSM	≥75 years vs. <70 years	N669 vs. N8268	48–49	Similar blood loss, 30 d complication rates, and urinary continence. Worse BCRFS and erectile function.

No.: number, pts.: patients, R: retrospective study, C: comparative study, IPTW: inverse probability of treatment-weighted study, PSM: propensity-score-matched study, BCRFS: biochemical recurrence failure-free survival, CSS: cancer-specific survival, OS: overall survival, ADT: androgen deprivation therapy, DM: diabetes, PCa: prostate cancer, BMI: body mass index, GS: Gleason score, RM: resection margin, sFI: simplified Frailty Index.

**Table 2:** Relationships between frailty and postoperative outcomes in radical prostatectomy.

First Author, Year	Surgical Approach	Type of Tools	No. Pts.	Findings
Levy, 2017 [77]	RARP	mFI-15	23,104	mFI-15 ≥ 3 showed OR 12.1 (CI: 2.8–52.4, *p* < 0.005) for Clavied–Dindo grade 4 complications
Rosiello, 2020 [78]	Miscellaneous	Johns Hopkins ACG	91,618	Higher rates of overall complications (16.6% vs. 8.6%) and major complications (4.9% vs. 2.6%)
Momota, 2020 [79]	RARP	G8 *, sFI, NRS	154	Frailty defined by both terms (G8 ≤ 14 or sFI 0–1) was not associated with postoperative complications, but frailty defined by G8 was associated with NRS ≥ 5 (moderate to severe pain)
Shahait, 2021 [80]	Miscellaneous	5-iFI	33,643	5-iFI ≥ 2 score was associated with higher Clavien–Dindo grade complications, longer length of stay, and increased risk of mortality
Togashi, 2021 [81]	RARP	G8 *	118	No association between frailty and health-related QUL or LUTS
Kodama, 2021 [82]	RARP	G8 *	479	Age, cerebrocardiovascular disease or chronic respiratory disease, and G8 scores were associated with surgical contraindications

No.: number; pts.: patients; RARP: robot-assisted radical prostatectomy; mFI-15: modified Frailty Index; OR: odds ratio; CI: confidence interval; ACG: adjusted clinical groups; sFI: simplified Frailty Index that consists of 4 comorbidities (hypertension, diabetes, cardiovascular disease, chronic respiratory disease) and instrumental activities of daily living; NRS: Numeric Rating Scale; 5-iFI: a total score was calculated by assigning a point for each conditions such as chronic obstructive pulmonary disease, congestive heart failure, dependent functional status, hypertension, and diabetes. * G8 ≤ 14 was used for cut-off in defining frailty.
